# AOP-helpFinder 3.0: from text mining to network visualization of key event relationships, and knowledge integration from multiple sources

**DOI:** 10.1093/bioinformatics/btaf381

**Published:** 2025-06-28

**Authors:** Thomas Jaylet, Florence Jornod, Quentin Capdet, Olivier Armant, Karine Audouze

**Affiliations:** Université Paris Cité, Inserm, HealthFex, Paris F-75006, France; Université Paris Cité, Inserm, HealthFex, Paris F-75006, France; Université Paris Cité, Inserm, HealthFex, Paris F-75006, France; PSE-ENV/SERPEN/LECO, Institut de Radioprotection et de Sûreté Nucléaire (IRSN), Saint-Paul-Lez-Durance, France; Université Paris Cité, Inserm, HealthFex, Paris F-75006, France

## Abstract

**Motivation:**

The Adverse Outcome Pathways (AOP) framework advances alternative toxicology by prioritizing the mechanisms underlying toxic effects. It organizes existing knowledge in a structured way, tracing the progression from the initial perturbation of a molecular event, caused by various stressors, through key events across different biological levels, ultimately leading to adverse outcomes that affect human health and ecosystems. However, the increasing volume of toxicological data presents a significant challenge for integrating all available knowledge effectively.

**Results:**

Text mining techniques, including natural language processing and graph-based approaches, provide powerful methods to analyze and integrate large, heterogeneous data sources. Within this framework, the AOP-helpFinder TM tool, accessible as a web server, was created to identify stressor-event and event-event relationships by automatically screening scientific literature in the PubMed database, facilitating the development of AOPs. The proposed new version introduces enhanced functionality by incorporating additional data sources, automatically annotating events from the literature with toxicological database information in a systems biology context. Users can now visualize results as interactive networks directly on the web server. With these advancements, AOP-helpFinder 3.0 offers a robust solution for integrative and predictive toxicology, as demonstrated in a case study exploring toxicological mechanisms associated with radon exposure.

**Availability and implementation:**

AOP-helpFinder is available at https://aop-helpfinder-v3.u-paris-sciences.fr.

## 1 Introduction

Human beings and ecosystems are exposed daily to an increasing number of potentially toxic substances and other factors (stressors), whose effects and risks are often poorly characterized, partly due to the low efficiency of traditional in vivo testing. Modern toxicology relies on new alternative approaches (NAMs) and high-throughput methodologies to address this limitation (Krewski *et al.* 2010, Krewski *et al.* 2020). In 2010, the concept of Adverse Outcome Pathways (AOPs), proposed by Ankley *et al.* emerged as an effective solution to integrate and structure existing toxicological knowledge, thus supporting mechanistic understanding and risk assessment in response to the growing demand for the evaluation of stressors (Ankley *et al.* 2010). An AOP describes a sequence of events that starts with a Molecular Initiating Event (MIE), triggered by a prototypical stressor. This MIE is linked to a sequence of key events (KEs) across different levels of biological organization, ultimately leading to an outcome (AO) at the individual or population level. These events (MIEs, KEs, AOs) are connected by key event relationships (KERs) that describe their causal links, enhancing the understanding of stressor(s) toxicity. Biological events can be shared across multiple AOPs, allowing the formation of Adverse Outcome Pathways Networks (AOPNs) that reflect the complexity of biological systems (Villeneuve *et al.* 2014, Yarar and Wojewodzic 2024).

Given the vast amount of available data, identifying and integrating relevant toxicological information into an AOP can be difficult and time-consuming. Text mining (TM) offers effective techniques to address these issues, and the AOP-helpFinder tool was developed with this objective in mind. Available as a web server, AOP-helpFinder combines TM, graph theory, and natural language processing (NLP) methods to identify relationships between stressors and biological events (MIE, KE, AO), as well as between pairs of biological events (KE-KE), by analyzing PubMed abstracts (method previously described in Carvaillo *et al.* 2019, Jornod *et al.* 2022, Jaylet *et al.* 2023). Confidence scores were assigned to each link based on co-occurrence frequency and statistical significance (Fisher’s exact test, as detailed in Jaylet *et al.* 2023), thereby contributing to the Weight of Evidence (WoE) evaluation.

However, it is crucial to consider data from multiple sources, such as toxicological databases to cross AOPs with more existing knowledge. Therefore, we present an enhanced version of AOP-helpFinder that automatically links and annotates extracted information from the literature to various relevant databases using a systems biology approach. This version also includes the capacity to visualize the identified KER as interactive biological networks directly from the web server. A case study is presented to demonstrate the tool's effectiveness, focusing on identifying and capturing potential mechanisms and pathologies induced after radon exposure, integrating scientific literature and multi-source databases knowledge.

## 2 Upgrades and new features

### 2.1 Knowledge annotation using multi-source databases

The AOP-helpFinder tool was developed to extract relevant relationships (between stressor and event, and between events) from PubMed abstracts, facilitating the establishment of AOPs. However, developing an AOP requires comprehensive toxicological information, which can also be stored in various databases. This enhanced version of the tool automatically annotates biological events (MIE, KE, AO) identified in the literature with information from open-access toxicological databases.

Among them, the AOP repository named AOP-Wiki, which directly links the identified events to existing AOPs, was integrated (https://aopwiki.org). Other databases provide complementary information, such as the Human Protein Atlas (HPA), which details gene expression in different tissues, various signaling pathways databases, e.g. KEGG, Reactome, WikiPathways, Uniprot for human gene-pathway/process associations, and for human gene-disease associations the DISEASES (JensenLab, knowledge section) and DisGeNET (curated section) databases were included. The annotation adopts a systems biology approach, providing information across multiple levels of biological organization. The tool automatically retrieves the latest versions of the databases, except for DisGeNET, which is based on a fixed version (see Section I, available as supplementary data at *Bioinformatics* online and Table 1, available as supplementary data at *Bioinformatics* online—10.5281/zenodo.15193935).

**Figure 1.**
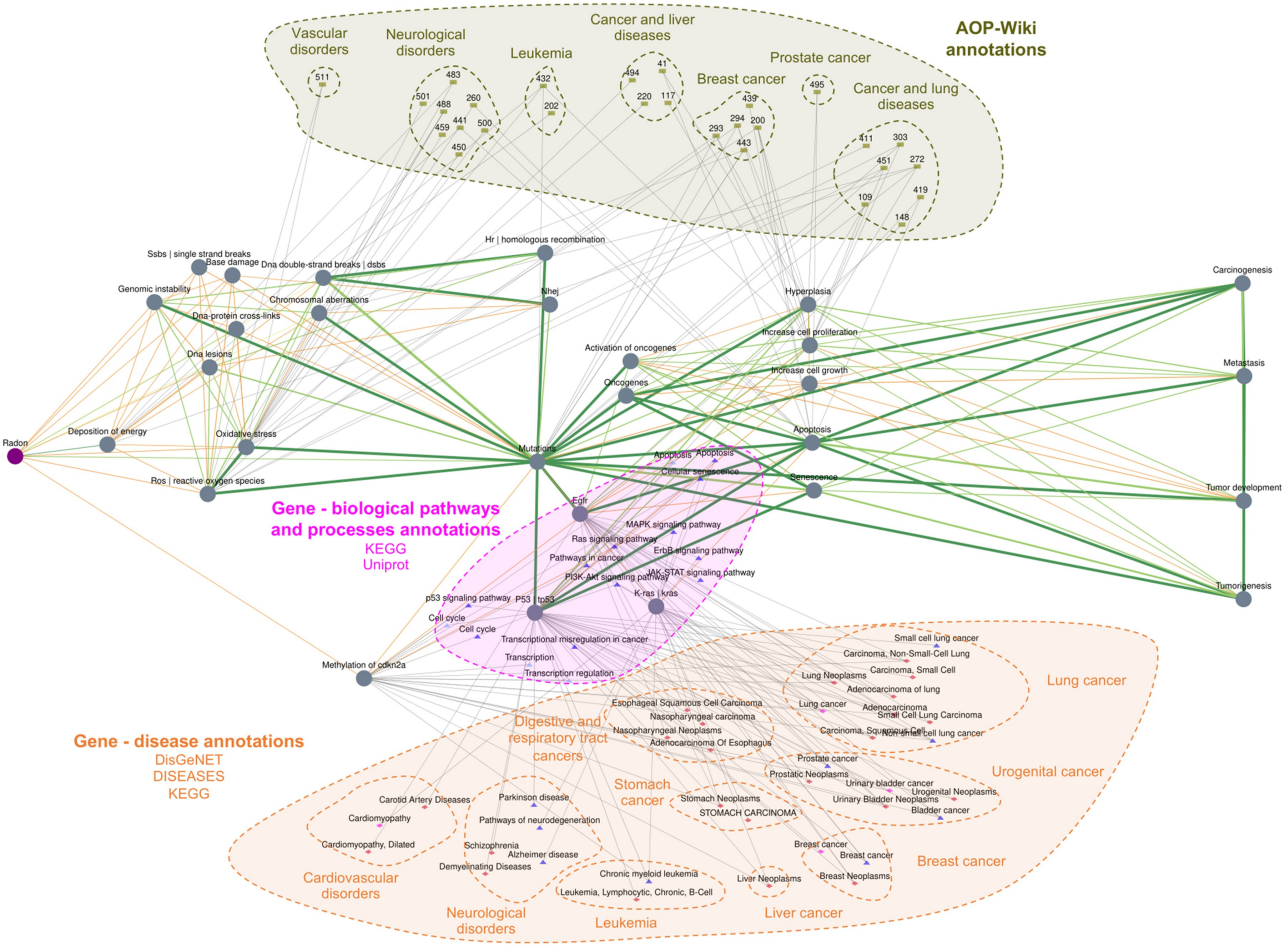
AOPN associated with radon exposure. The round nodes represent the stressor (purple) and biological events (gray) extracted from PubMed literature using TM, connected by edges weighted according to the number of articles addressing the link, and colored based on the confidence score (orange: Low; yellow: Moderate; light green: High; dark green: Very High). Annotations from databases are surrounded by dashed lines: AOPs in khaki, gene-disease annotations in orange, and gene-pathway annotations in magenta. The network is undirected and represents associations extracted from both the literature and external databases.

Since AOP-helpFinder searches for links based on user-provided inputs and given that the information in the databases may not be uniformly formatted, data standardization is performed to ensure efficient and accurate automatic annotation. This standardization is achieved through NLP methods (Python; NLTK v. 3.8.1), including stop word removal, stemming, and the consideration of synonyms (e.g. different synonyms for the same biological event or gene) (see Section II, available as supplementary data at *Bioinformatics* online and Fig. 1, available as supplementary data at *Bioinformatics* online—10.5281/zenodo.15193935).

### 2.2 Interactive visualization in the form of biological networks

AOP-helpFinder 3.0 offers visualization of all findings generated by the method as biological networks. The networks are integrated into a web page specific to each user and uses the Cytoscape.js package (v. 3.19.1) and JavaScript, allowing the opening and processing of data from a .json file containing network information (nodes, edges, descriptions, annotations).

The default settings display all associations extracted from the literature by AOP-helpFinder, with nodes representing biological events or stressors and edges representing the relationships. Edges are weighted based on the number of links and colored according to the confidence score assigned to each association (Fig. 2, available as supplementary data at *Bioinformatics* online) (see details of the confidence in Jaylet *et al.* 2023). Users can click on an edge to display a table containing all PubMed articles related to the association (Fig. 3, available as supplementary data at *Bioinformatics* online). The network also allows filtering results based on the number of PubMed links extracted by AOP-helpFinder and/or the confidence score assigned to these links (from the “main menu” tab—Fig. 4, available as supplementary data at *Bioinformatics* online). Additionally, it enables displaying annotations for individual events (by clicking on a node) or for the entire network (from the “main menu” tab) provided by the different databases (Figs 4–7, available as supplementary data at *Bioinformatics* online).

The generated network can be saved as an image (.png or high-quality vector image .svg), and users can export all information as a table (.tsv) readable in Cytoscape, allowing for more advanced analysis of the results. The network features a “Help” tab (?), containing a description of all functions (Fig. 2, available as supplementary data at *Bioinformatics* online).

### 2.3 Redesign of the web server

Moreover, the AOP-helpFinder web server has been redesigned with a more modern and user-friendly interface, adding new functionalities. The homepage now offers two distinct options: (i) searching via AOP-helpFinder and (ii) visualization of previous results as networks (Fig. 8, available as supplementary data at *Bioinformatics* online). The AOP-helpFinder search now includes new options, allowing users to choose between the original TM method (which requires finding at least ¾ of the event’s words to consider a link in the literature) (Jaylet *et al.* 2023) and a more precise but restrictive method that requires finding all words, thereby reducing the risk of false positives. The tool also allows for combined stressor-event and event-event searches, enabling the generation of a computational pre-AOP (or AOPN) and linking stressors to this pre-AOP. This TM step is then automatically complemented by annotating the biological events with database information. This search requires the user's mail address to access the upload page and to receive notifications when results are available for download or network visualization. As with previous versions, all results and mail addresses are automatically deleted after one month, ensuring privacy and aligning with digital sobriety to reduce environmental impact by limiting computing use.

## 3 Case study: developing a computational AOPN induced by radon exposure

The set of exposures an individual encounters throughout his life is conceptualized as the exposome (Wild 2005). Many different components represent the exposome, such as chemicals, including pollutants, pesticides, additives, and drugs, as well as physical factors such as radiation emitted by radon, which can pose significant challenges to human health and ecosystems. Chronic residential exposure to radon is currently considered the second leading cause of lung cancer worldwide, after tobacco, and the primary cause among non-smokers (WHO 2009). Epidemiological studies have also suggested radon's involvement in extra-pulmonary diseases, such as neurodegenerative diseases (Lehrer *et al.* 2017), stomach cancers (Barbosa-Lorenzo *et al.* 2017) and an increased risk of leukemia (Rericha *et al.* 2006, Lu *et al.* 2020). Although the majority of inhaled radon deposits in the lungs and irradiates lung tissue, studies have shown that a small fraction can enter systemic circulation and distribute to various tissues, including bone marrow, affecting hematopoietic stem cells and potentially explaining observations regarding leukemia (Richardson *et al.* 1991, Sakoda *et al.* 2010), as well as the brain, affecting radiosensitive glial cells and contributing to neurodegenerative disorders (Zhang *et al.* 2022). Additionally, radon, found naturally in the environment, can also deposit in water, potentially posing a risk for stomach cancers (Barbosa-Lorenzo *et al.* 2017). However, while residential radon exposure seems to induce extra-pulmonary effects, none of these effects have been causally demonstrated, and the mechanisms associated with these pathologies remain undefined.

To gain a comprehensive understanding of the mechanisms and risks associated with radon exposure, we took advantage of the AOP framework. We conducted a combined “stressor–event” and “event–event” search using AOP-helpFinder 3.0 to automatically identify relationships between radon and a list of 41 biological events, including genes previously identified by experts as potentially associated with radon exposure (Table 2, available as supplementary data at *Bioinformatics* online). This TM step, based on PubMed abstracts, enabled the construction of a preliminary radon–event–event network, in which 28 of the 41 selected events were directly linked to radon. These events were then automatically annotated using toxicological databases integrated into AOP-helpFinder 3.0. In total, more than 800 biological annotations were retrieved, enriching the initial literature-based network with information related to AOPs, diseases, and biological pathways. The unfiltered network is shown in Fig. 9, available as supplementary data at *Bioinformatics* online, and the complete dataset as well as all steps necessary to reproduce this case study are available in the Supplementary Materials (10.5281/zenodo.15193935). The annotations produced informative results. For example, AOP-Wiki annotations linked the extracted events to several AOPs related to cancer (lung, breast, urogenital, liver, leukemia) and to other conditions such as neurological and vascular disorders, thus providing potential mechanistic insights. Moreover, gene annotations from KEGG, Reactome, WikiPathways, DisGeNET, and DISEASES databases corroborated these associations and helped to validate the findings. These annotations also highlighted key biological pathways potentially involved in radon-related toxicity, including cell cycle disruption, apoptosis, and transcriptional dysregulation, particularly involving TP53, as well as additional pathways relevant to cancer and neurological diseases.

After filtering to retain the most informative connections, the network presented in Fig. 1 was obtained. It displays the results as a fully computational AOPN, emphasizing the most relevant annotations for this case study. In summary, this computational AOPN, integrating literature information and annotations from databases, presents consistent results and demonstrates that radon primarily exerts its deleterious effects through DNA damage, promoting mutations (e.g. TP53, KRAS, EGFR) and alterations in gene expression (e.g. CDKN2A) involved in key pathways such as apoptosis and the cell cycle, leading to disease development. Notably, these toxic mechanisms appear to be similar across different diseases. Although these predictive results should be interpreted with caution, this case study illustrates the potential of AOP-helpFinder 3.0 to integrate relevant information from multiple sources, providing a starting point for future research and supporting modern, predictive toxicology.

## 4 Conclusion

AOP-helpFinder 3.0 is a powerful, interactive, and user-friendly tool designed to efficiently identify, extract, and prioritize biological knowledge. By conducting an in-depth screening of PubMed abstracts and annotating relevant information with complementary toxicology data from multiple databases, it enables rapid extraction of relevant knowledge. Results can be viewed directly on the web server as comprehensive biological networks, facilitating the development of AOPs and AOPNs and providing a platform that supports the expert curation still needed to ensure the accuracy and biological relevance of the data mining results. This tool supports next-generation risk assessment by incorporating mechanistic insights and helps pinpoint knowledge gaps, guiding the direction of future research efforts. Looking ahead, integrating systematic quality assessment strategies, such as community-driven evaluations or filtering of low-quality publications, could enhance the reliability of the extracted links and further strengthen the scientific foundation of the networks.

## Supplementary Material

btaf381_Supplementary_Data

## Data Availability

All figures, supplementary figures, and files used for this study are available on Zenodo 10.5281/zenodo.15193935. The scripts are available on https://github.com/systox1124/AOP-helpFinder.
