# Supplementary material for: AOP-helpFinder 3.0: from text mining to network visualization of key event relationships, and knowledge integration from multiple sources
Source: Bioinformatics. 2025 Jun 28;41(7):btaf381. doi: 10.1093/bioinformatics/btaf381 (PMC12263105; doi:10.1093/bioinformatics/btaf381)
Supplement: btaf381_Supplementary_Data [file btaf381_supplementary_data.docx]

**Supplementary Material**

**AOP-helpFinder 3.0: from text mining to network visualization of key event relationships, and knowledge integration from multiple sources**

Thomas Jaylet, Florence Jornod, Quentin Capdet, Olivier Armant, Karine Audouze

**Table of contents**

[**I - Description of the databases used for annotation 2**](#_heading=h.y1w7bu6y6nu3)

[**II - Annotation of knowledge from the literature using multi-source databases and Natural Language Processing (NLP) techniques 4**](#_heading=h.mbxou9xo8wgt)

[**III – Biological network provided by AOP-helpFinder 3.0 6**](#_heading=h.j40gh1zfuw60)

[**IV – AOP-helpFinder 3.0 web server 9**](#_heading=h.odyym57yi9hr)

[**V – Case study: AOP network induced by radon exposure 10**](#_heading=h.2lcnjv73xa4t)

[*Figure S 1: Example of the importance of NLP and synonym consideration in data standardization for annotation with databases. 5*](#_heading=h.r5o10y2ar9aq)

[*Figure S 2: AOP-helpFinder network - Design and default parameters of the biological network. 6*](#_heading=h.jpii91z8ob5g)

[*Figure S 3: AOP-helpFinder network – Edge information. 6*](#_heading=h.uwwydn1z3q7i)

[*Figure S 4: AOP-helpFInder network - Options in the main menu. 7*](#_heading=h.tmrqo1ej007p)

[*Figure S 5: AOP-helpFinder network – Node annotation. 7*](#_heading=h.rkdrqsgwo8jd)

[*Figure S 6: AOP-helpFinder network - Annotation panel. 8*](#_heading=h.a83eu2iosszv)

[*Figure S 7: AOP-helpFinder network - Information on annotations. 8*](#_heading=h.8vzcutdk600x)

[*Figure S 8 : AOP-helpFinder web server – new design for version 3. 9*](#_heading=h.jro7g5nmjoj)

[*Figure S 9: Unfiltered raw network provided by AOP-helpFinder 3.0. 11*](#_heading=h.kc2jf1jpqmdu)

[*Table S 1: Information on all toxicological databases used for the annotation of AOP-helpFinder events. 3*](#_heading=h.j775pci4pzym)

[*Table S 2: List of the 41 events searched by AOP-helpFinder. 10*](#_heading=h.6eydoign7ijw)

# I - Description of the databases used for annotation

Annotation is performed using various open access databases. AOP-Wiki allows for the annotation of complete biological events (e.g., ‘Increased oxidative stress’, ‘Lung cancer’) and their linkage to AOPs. Other databases, such as those for biological pathways (KEGG, Reactome, WikiPathways, Uniprot), diseases (DisGeNET, DISEASES), and gene expression profiles (Human Protein Atlas), enable the annotation of biological events involving genes (e.g., ‘TP53 mutation’, ‘AHR activation’). The different databases and the role of annotation are described below. Table S1 summarizes essential information about annotation and the versions used.

**AOP-Wiki**: AOP-Wiki (<https://aopwiki.org/>) is a collaborative platform that provides detailed information on Adverse Outcome Pathways (AOPs). It supports the development and dissemination of AOP knowledge by offering a structured format for describing mechanistic links between molecular, cellular, and tissue events and adverse health effects.
Annotation with AOP-Wiki links the biological events identified by AOP-helpFinder to existing AOPs, providing a comprehensive view of the toxicological mechanisms associated with an event.

**KEGG**: The Kyoto Encyclopedia of Genes and Genomes (KEGG) (<https://www.genome.jp/kegg/>) is a comprehensive database resource for understanding high-level functions of biological systems.
Annotation with KEGG provides information on gene functions, biological pathways, and diseases.

**Reactome**: Reactome (<https://reactome.org/>) is an open-source, open-access, manually curated, and peer-reviewed pathway database.
Annotation with Reactome provides detailed information about various biological pathways, including metabolic pathways, signal transduction pathways, and pathways involved in the regulation of cellular processes.

**WikiPathways**: WikiPathways (<https://www.wikipathways.org/>) is an open, collaborative platform dedicated to the curation and annotation of biological pathways.
Annotation with WikiPathways provides insights into metabolic, signaling, and regulatory pathways by linking genes and proteins to specific biological processes and events.

**DisGeNET**: DisGeNET (<https://www.disgenet.org/>) is a database containing one of the largest publicly available collections of genes and variants associated with human diseases. It integrates data from various sources, including curated data, data from animal models (using orthology information to map associations to human genes), inferred data, and literature data.
Annotation with DisGeNET is used to link curated data between genes and diseases. *Annotation is based on the 2015 version, as newer versions do not allow easy, open-access download of direct gene-disease association data.

**DISEASES (JensenLab)**: DISEASES (<https://diseases.jensenlab.org/>) is a database that integrates evidence on disease-gene associations from automatic text mining, curated data (knowledge), and experimental data.
Annotation provides a comprehensive overview of curated gene-disease associations, supporting the understanding of disease mechanisms.

**The Human Protein Atlas (HPA)**: The Human Protein Atlas (<https://www.proteinatlas.org/>) is a resource that provides information on the tissue and cellular distribution of all human proteins, as well as information on human gene expression profiles.
Annotation provides insights into the expression profiles of genes at the mRNA level in human tissues.

**UniProt**: UniProt (Universal Protein Resource) is a comprehensive resource that provides a central repository of protein information, including function, domain structure, post-translational modifications, variants, and involvement in diseases.
Annotation with UniProt informs about the biological processes in which proteins (genes) are involved. *Data on biological processes in UniProt are retrieved from HPA.

***Table S 1: Information on all toxicological databases used for the annotation of AOP-helpFinder events.***

The table includes the type of annotations, the version used*, and links for data retrieval. *Except for DisGeNET, the latest version of each database is automatically retrieved, ensuring that the annotation is performed with the most recent data.

|  | AOP-Wiki | HPA | KEGG | Reactome | Uniprot (from HPA) | WikiPathways | DisGeNET (curated) | DISEASES (knowledge) |
| --- | --- | --- | --- | --- | --- | --- | --- | --- |
| Annotation | AOPs | Gene expression in human tissues | Human genes – biological pathways/processes | | | | Human genes – diseases | |
| Database version | Latest  (v 2.7) | Latest  (v 23.0) | Latest  (v 110.0) | Latest  (v 88) | Latest HPA version  (v 23.0) | Latest (20240710) | 05.2015  (v 3.0) | Latest  (v 2.0) |
| Accessibility |  | Open access | | | | | | |
| Download | <https://aopwiki.org/downloads/aop_ke_mie_ao.tsv> | <https://www.proteinatlas.org/download/proteinatlas.tsv.zip> | Via R package  (KEGG REST) | <https://reactome.org/download/current/ReactomePathways.gmt.zip> | <https://www.proteinatlas.org/download/proteinatlas.tsv.zip> | <https://data.wikipathways.org/current/gmt/wikipathways-20241010-gmt-Homo_sapiens.gmt> | DisGeNET curated gene-diseases associations dataset (05.2015) | <https://download.jensenlab.org/human_disease_knowledge_filtered.tsv> |

# II - Annotation of knowledge from the literature using multi-source databases and Natural Language Processing (NLP) techniques

AOP-helpFinder searches for links based on user-provided inputs. Consequently, the data retrieved by AOP-helpFinder and those from databases may not be uniformly formatted, necessitating standardization to account for various nomenclatures and correct manual errors.

For example, if a user searches for the event 'Increased oxidative stress' to find associations with stressors of interest (stressor-event) or with other biological events (event-event) in the literature, AOP-helpFinder will then seek to add additional information through annotation with databases. For instance, it will search for a corresponding KE in AOP-Wiki to identify all AOPs containing this event. In this case, the AOP-Wiki KE 1088 is titled ‘Increase, Oxidative stress’. Despite being similar, the terms ‘Increased oxidative stress’ and ‘Increase, oxidative stress’ cannot be automatically connected due to slight terminological differences (conjugation and punctuation) (Figure S1). To ensure maximum connectivity, natural language processing (NLP) methods, including stopword removal and stemming, are employed (Python, NTLK v 3.8.1). Stemming retains only the stem or root of words, removing suffixes, while stopwords refer to non-essential words for text comprehension, such as conjunctions (but, or, and...), determiners (the, a, of...), and punctuation (;?!), which are removed from sentences. Thus, applying NLP eliminates variations in conjugation and punctuation. In the previous example, ‘Increased oxidative stress’ and ‘Increase, oxidative stress’ become ‘increas oxid stress’ and can thus be connected (Figure S1).

Another challenge for automatic connection between AOP-helpFinder events is different nomenclatures/synonyms for the same term. For instance, if a user searches for ‘Reactive oxygen species’ with AOP-helpFinder, it will not be annotated with KE 1278 ‘ROS formation’ (Figure S1). Similarly, some genes are commonly written with unofficial spellings. For example, if a user searches for the event ‘p53’ on AOP-helpFinder, it will not be annotated by pathway (KEGG, Reactome, etc.) and disease (DISEASES, DisGeNET) databases, as its official nomenclature is ‘TP53’. Therefore, in addition to NLP methods, the tool uses a list of common biological event synonyms from AOP-Wiki (e.g., ROS: Reactive oxygen species; ER: Estrogen receptor; IL-6: Interleukin-6, etc.), as well as a list of gene synonyms (official and unofficial nomenclature; e.g., TP53, P53, LFS1) referenced in the HPA database. This step ensures that essential information is not lost. For instance, ‘Reactive oxygen species’ can be automatically connected to KE 1278 ‘ROS formation’. Similarly, if a user searches for an event including P53, it will be annotated by databases referencing it under its official symbol ‘TP53’ (Figure S1).

Finally, if a user searches for a broad or imprecise event with AOP-helpFinder (e.g., ‘Cancer’), it will be annotated with a set of more specific events on AOP-Wiki, such as KE 1193 ‘Breast cancer’ and 1670 ‘Lung cancer’, ensuring the retrieval of maximum biological information, while ensuring that the reverse is not possible to avoid introducing unnecessary noise.

***Figure S 1: Example of the importance of NLP and synonym consideration in data standardization for annotation with databases.***

NLP*: Natural Language Processing; KEGG*: Similarly applied to KEGG, Reactome, WikiPathways, DISEASES, DisGeNET, HPA (+ Uniprot) databases.

# III – Biological network provided by AOP-helpFinder 3.0

This section presents the design and features of the result visualization tool integrated into AOP-helpFinder 3.0, which displays data as a biological network.

***Figure S 2: AOP-helpFinder network - Design and default parameters of the biological network.***

*The round nodes represent the stressor (purple) and biological events (gray) extracted from PubMed literature using text mining, connected by edges weighted according to the number of articles addressing the link, and colored based on the confidence score.*

*The navigation bar includes various functionalities:* ***Help**** *(opens a file with information),* ***Full screen (+/-)**** *(toggles full screen mode),* ***Zoom (+/-)**** *(zooms in or out),* ***Default settings**** *(resets to default parameters),* ***Undo**** *(reverts the last change),* ***Delete**** *(removes selected nodes or edges),* ***Save*** *(opens a panel to save results as a table or image (.png or .svg)),* ***Legend**** *(opens a panel showing the different legends), and* ***Main menu**** *(offers options for filtering results and annotating the entire network).*

***Figure S 3: AOP-helpFinder network – Edge information.***

*Clicking on an edge connecting two nodes (stressor-event or event-event) displays all articles related to the relationship identified by AOP-helpFinder 3.0.*

***Figure S 4: AOP-helpFInder network - Options in the main menu.***

***Filtering**** *by confidence score or number of links.* ***Layouts**** *to change the network's layout.* ***Statistics**** *to provide information on the number of nodes and links.* ***Network annotation*****, which opens the annotation panel for each database (AOP-Wiki, Uniprot, KEGG, Reactome, WikiPathways, DISEASES, DisGeNET, HPA).*

***Figure S 5: AOP-helpFinder network – Node annotation.***

*Clicking on a node opens an information panel, allowing the annotation of that node with different databases. *Reactome and WikiPathways are not displayed in this figure.*

***Figure S 6: AOP-helpFinder network - Annotation panel.***

*Example for the node "Deposition of Energy" and the AOP-Wiki database. The principle is the same for annotation through the main menu, with the difference that it will be possible to display all annotations simultaneously, not just those of the selected node. The results are sorted according to their degree. The Degree column corresponds to the number of connections. For example, AOP 272, with a degree of 3, is connected to 3 distinct network events.*

***Figure S 7: AOP-helpFinder network - Information on annotations.***

*After displaying an annotation, clicking on a node opens a table showing the information related to that annotation. This figure shows the example for the annotation with AOP-Wiki for AOP 272, indicating all its components (MIE, KE, AO).*

# IV – AOP-helpFinder 3.0 web server


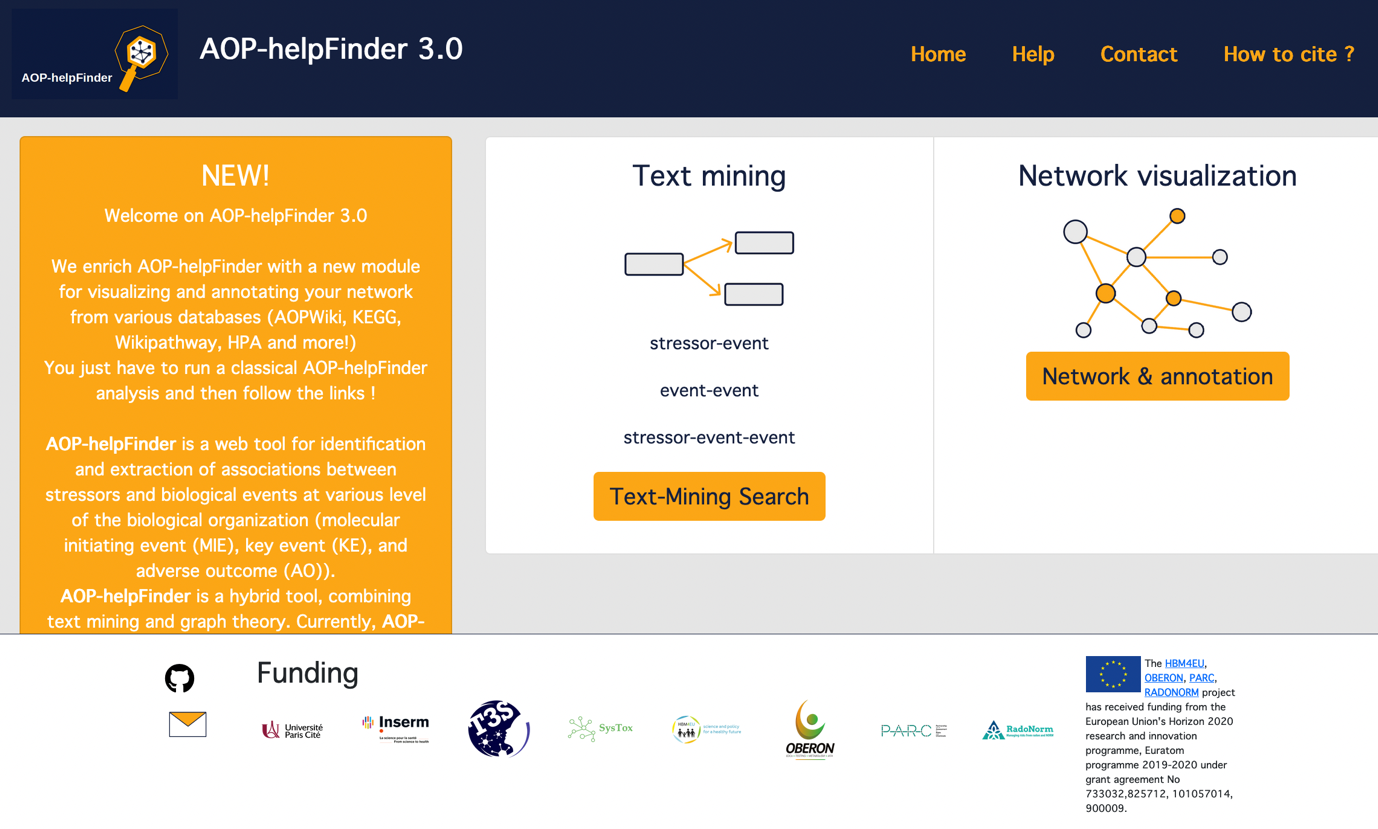


***Figure S 8 : AOP-helpFinder web server – new design for version 3.***

*The tool offers the possibility to: (1) perform a search (stressor-event, event-event, or both), resulting in downloadable and visualizable results in the form of networks (left). (2) Re-visualize a previous result by providing the search ID that was given (right). AOP-helpFinder can sometimes take a long time to run. To alleviate this issue, we have considerably increased our server capacities (20 CPU to 102 CPU). However, in order to speed up the search, the user can select more precise words to process fewer articles. For example, instead of searching for “cancer” as a stressor, one will search for “breast cancer”, which will reduce the number of articles from > 5,000,000 to > 500,000*

# V – Case study: AOP network induced by radon exposure

***Table S 2: List of the 41 events searched by AOP-helpFinder.***

*This list of events was manually curated by identifying events potentially associated with radon or ionizing radiation from the AOP-Wiki database and scientific literature. The* ***28 events*** *in bold correspond to those for which AOP-helpFinder 3.0 found an association with radon through text mining (stressor-event) and were subsequently used for an event-event search to establish the AOP network. The* ***15 events*** *in bold and orange are those that had at least one annotation from one of the various databases (AOP-Wiki, KEGG, Uniprot, DisGeNET, DISEASES, HPA).*

| Deposition of Energy |
| --- |
| Cascade of ionization events |
| Reactive oxygen species \| ROS |
| Oxidative stress |
| DNA lesions |
| DNA Double-Strand breaks \| DSBs |
| Single strand breaks \| SSBs |
| Base damage |
| DNA-DNA cross links |
| DNA-protein cross-links |
| Multiples damages sites |
| Clastogenic lesions |
| Genomic instability |
| Repair machinery damage |
| Inadequate DNA repair |
| Unrepaired DSBs |
| Chromosomal aberrations |
| NHEJ |
| Homologous Recombination \| HR |
| Apoptosis |
| Senescence |
| Death regulation |
| Increase cell growth |
| Cell cycle dysregulation |
| Increase Cell proliferation |
| Mutations |
| KRAS \| K-RAS |
| EGFR |
| TP53 \| P53 |
| Methylation of CDKN2A |
| Lethal damage |
| Tumorigenic pathway |
| Hyperplasia |
| Formation of neoplasms |
| Tumor development |
| Metastasis |
| Activation of oncogenes |
| Inactivation of tumor-suppressor |
| Oncogenes |
| Carcinogenesis |
| Tumorigenesis |


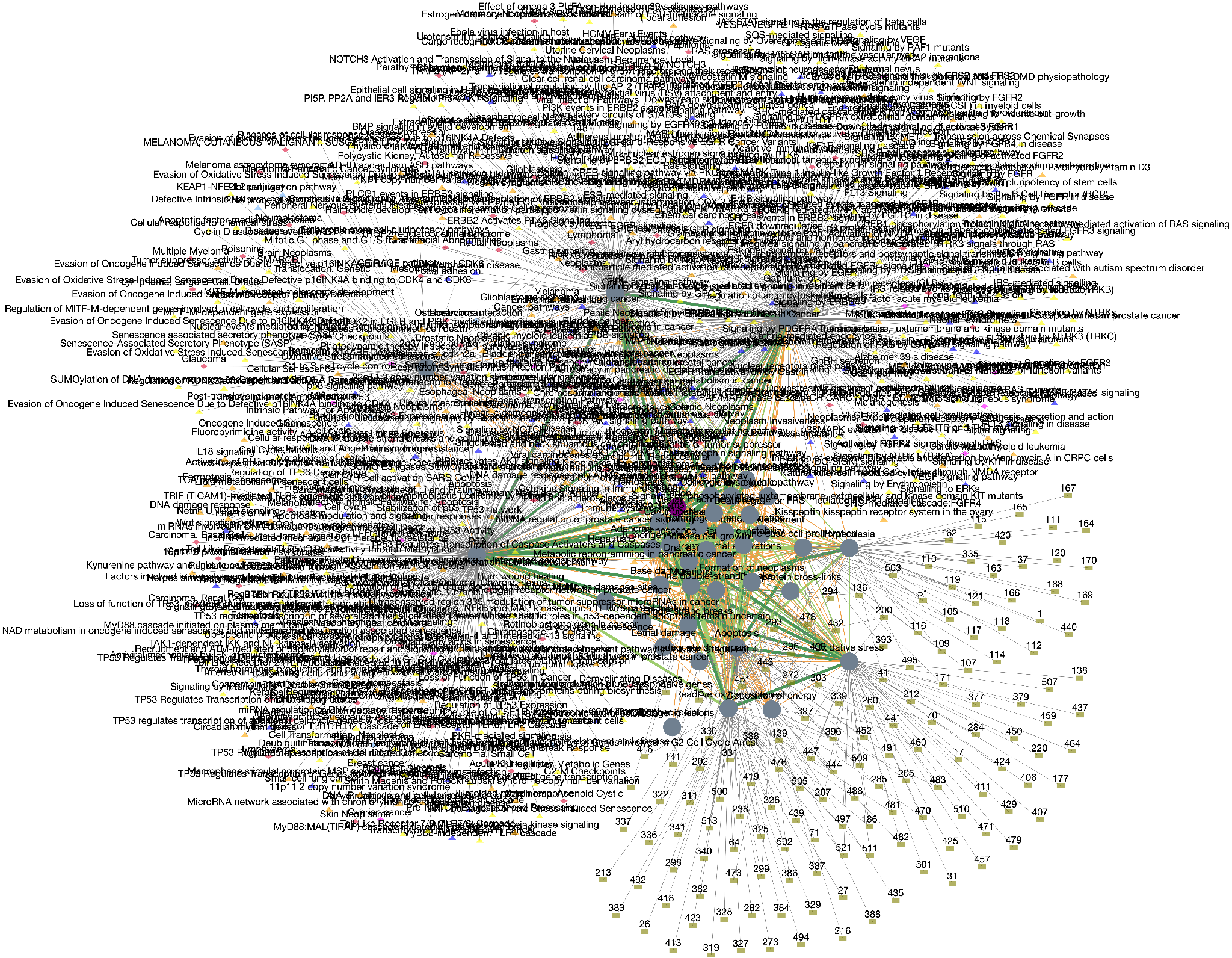


***Figure S 9: Unfiltered raw network provided by AOP-helpFinder 3.0.***

*This network includes all stressor-event and event-event links, as well as all biological annotations from AOP-Wiki (149), KEGG (107), Reactome (315), WikiPathways (148), Uniprot (9), DisGeNET (104) and DISEASES (16), totaling 832 annotations. All data used to generate this network, along with the Cytoscape-compatible file, are available in the supplementary Zenodo repository (*[*10.5281/zenodo.15193935*](https://doi.org/10.5281/zenodo.15193935)*.).*
